# The Mineral Waters of St. Catherines, Canada West

**Published:** 1858-08

**Authors:** 


*The Mineral Waters of St. Catherines, Canada West.*—There is a class of invalids to whom change of air and scene are absolutely necessary, and especially, removal from the cares of active life. Of all cares which oppress them, the cares of a family and a home are the most onerous when the mind is brooding over any real or fancied illness. Our female patients are especially susceptible to these influences, particularly those who are the subjects of uterine disease. To give relief to such sufferers, as well as to furnish a pleasant place of resort for those who are not afflicted, we have innumerable watering places, the very name of which is suggestive of the peculiar mode of life which is enjoyed by the *sardine* in his comparatively commodious tin case. This presents a fearful prospect to the meek *pater familias* who may have the temerity to suggest that an individual weighing two hundred pounds requires a certain quantity of space and a good proportion of oxygen, in order to sustain life through the eight hours which are allotted to sleep, and also that as "continual dropping wears away stone," so the persistent efforts of the active mosquito and the more stealthily, though not less terrible, *Acanthia Lectularia* (*Anglais, bed-bug*) will have an appreciable effect upon the above mentioned two hundred pounds. In spite of all these terrors, crowds of people yearly spend their summers at watering places, where they "suffer and are strong," that is, stronger than they would be at home.

This is decidedly the dark side of the picture, and physicians have always been disposed to attribute any benefit which is derived from a visit to a medicinal spring, to freedom from care, change of scene, air, etc., and not to the virtues of the water. There are, in Europe, however, waters which are acknowledged by the highest authorities to possess decided medicinal qualities, and whose curative powers in certain diseases, are beyond question. An analysis of the St. Catherines' waters, with remarks on their curative qualities, appeared in the January number of the New York Journal of Medicine, and a similar article was published in the number of the Buffalo Medical Journal for Sept., 1856, snowing a great similarity between this spring and the European spas which enjoy such a wide-spread reputation.

We copy the qualitative analysis by Dr. Mack, and the quantitative analysis by Prof. Henry Croft, of Toronto.

QUALITATIVE ANALYSIS. Taste intensely saline, giving the smell of sea-water. Neutral to test paper. Sp. gr., 1036. Temp. 60°; temp. of air 67° Fahr.

*Results of Analysis.*

Chloride of Sodium.
" " Calcium.
" " Magnesium.
" " Soda.
Iodide of Magnesium.
Bromide of Magnesium.
Carbonic Acid.
Alumina.
Silica.
Organic Matter.

QUANTITATIVE ANALYSIS OF PROF. HENRY CROFT.

| | In 1.000 | In Pint, 7,680 gr. |
|---|---|---|
| Sulphate of Lime, . . . | 2.1923 | 16.8368 |
| Chloride of Calcium, . . . | 14.8544 | 115.0818 |
| " " Magnesium, . . | 3.3977 | 26.0944 |
| Iodide of Magnesium, . . | 0.0042 | 0.0322 |
| Bromide of Magnesium, . | a trace. | |
| Chloride of Potassium, . . | 0.3555 | 2.7302 |
| " " Sodium, . . | 29.8034 | 228.8901 |
| | | 388.6655 |
| " " Ammonium, } . . | a trace. | |
| Silicic Acid, } . | 50.6075 | |
| Loss, . . | 1.0670 | |
| | 51.6745 | |

The following is an analysis of the mother water by Prof. CHILTON, of New York. Sp. gr. at 60° Fahr. Quantity examined, one pint.

| | Grains, |
|---|---|
| Chloride of Calcium, . . . . . . | 2,950.40 |
| " " Magnesium, . . . . . . | 1,289.76 |
| " " Sodium, . . . . . . | 781.36 |
| Proto-chloride of Iron, . . . . . . | 13.76 |
| Sulphate of Lime, . . . . . . | 16.32 |
| Carb. of Lime and Magnesia, . . . . . | 2.08 |
| Iodide of Magnesium, . . . . . . | 2.11 |
| Bromide of Magnesium, . . . . . . | 2.01 |
| Silica and Alumina, . . . . . . | 2.47 |
| Total grains, . . . . . | 5,060.27 |

It is unnecessary to give the analysis of the Kruznach mother water or other springs, as given by Dr. Mack in his article in the New York Journal; it is sufficient to say that in the most important particulars, the St. Catherines water resembles closely these celebrated springs, which have been recommended by men of no less eminence than Prof. Locock, and Simpson of Edinburgh, and the highest authorities in Paris. In looking at the analysis of Prof. Croft, we cannot but be struck with the pains which nature has taken in making up her alterative prescription. It is composed of the same ingredients as those which are now embodied in the prescription of the enlightened practitioner. The iodides, bromides, and chlorides, are now established as the most reliable remedies in the diseases which are so much benefited by these waters, and nature certainly can here claim priority over science, as it has furnished for thousands of years, what science has only just discovered.

Dr. Mack very properly remarks that it is necessary to take the baths and drink the water in an intelligent manner, and to associate with it a suitable regimen and course of exercise. The diseases, also, to which such treatment is applicable will suggest themselves to every physician. Among the first, we place the gouty and rheumatic diathesis. We had an opportunity of witnessing the good effects of the waters upon such difficulties, in a recent visit to the spa, and are compelled to say that they are such as we rarely produce by artificial combinations of similar drugs. Cutaneous affections requiring constitutional treatment, and tumors of every description, which we can hope to disperse by the internal use of remedies, are immensely benefited by a judicious course of the waters. Lastly, uterine diseases are very much benefited, and we conceive, especially by the baths. The practitioner will readily see that we have mentioned but a few of the diseases to which such a course of treatment applicable, and will be able to supply those which we have omitted; as we did not intend to go into a minute account of all maladies which are advantageously treated by the iodides, bromides, chlorides, &c.

The effect of the internal exhibition of the St. Catherines water is of course variable in degree in different persons; it always, however, has a cathartic and diuretic effect; and we are assured by Dr. Mack, that the concentrated water, properly diluted, is one of the most certain diuretics he has ever employed. The beneficial influence of the water is greatly increased at times by a judicious use of other remedies in connection with it: especially those of a tonic or alterative character as, for example, ferruginous or iodurretted preparations. Dr. Mack also gives a favorable account of the joint use of the oleum morrhuæ. The baths are constantly used, and at a variety of temperatures, from the very hot to the cold bath at the temperature at which the water is drawn from the well. The hot bath is exceedingly stimulating in its effects, and is not followed by sedation, and the tepid and cold baths are peculiarly refreshing, and soon followed by the specific effects of the waters.

With such testimony to the beneficial influence of the water, and a knowledge of its chemical composition, we cannot but believe in its good effects, and from personal observation we can say, that in the classes of diseases to which it is applicable, we are certain that it is of great benefit.

In concluding our article on the medical properties of the water, a subject which should receive most careful consideration at the hands of the scientific man, we must say a word in regard to the facilities which are provided for its use, especially as at the beginning of the article we give vent to our indignation at the manner in which one's sense of comfort is frequently outraged at watering places. It would hardly pay to leave a comfortable dwelling in the city or country for a room six by six and three companions, to say nothing of invertebrata, to be able to drink the elixir of life. The proprietor of the St. Catherines well has fitted up a commodious bathing-house, and erected a comfortable hotel, presenting as striking a contrast as possible, to the picture we have drawn. With all these advantages, we know of no place where sufferers from gout, rheumatism, &c., could be more benefited, and we considered it our duty to let our readers know of its existence and advantages.

